# Mechanisms behind Temsirolimus Resistance Causing Reactivated Growth and Invasive Behavior of Bladder Cancer Cells In Vitro

**DOI:** 10.3390/cancers11060777

**Published:** 2019-06-04

**Authors:** Eva Juengel, Iyad Natsheh, Ramin Najafi, Jochen Rutz, Igor Tsaur, Axel Haferkamp, Felix K.-H. Chun, Roman A. Blaheta

**Affiliations:** 1 Department of Urology, Goethe University Hospital, 60590 Frankfurt am Main, Germany; ramin_n@hotmail.de (R.N.); Jochen.Rutz@kgu.de (J.R.); Felix.Chun@kgu.de (F.K.-H.C.); blaheta@em.uni-frankfurt.de (R.A.B.); 2 Department of Urology and Pediatric Urology, University Medical Center Mainz, Langenbeckstr. 1, 55131 Mainz, Germany; igor.tsaur@unimedizin-mainz.de (I.T.); axel.haferkamp@unimedizin-mainz.de (A.H.); 3 Department of Allied Medical Sciences, Zarqa University College, Al-Balqa Applied University, Salt 13110, Jordan; iyadnatsheh@bau.edu.jo

**Keywords:** bladder cancer, mechanistic target of rapamycin (mTOR), temsirolimus-resistance, growth, invasion, integrins

## Abstract

Background: Although mechanistic target of rapamycin (mTOR) inhibitors, such as temsirolimus, show promise in treating bladder cancer, acquired resistance often hampers efficacy. This study evaluates mechanisms leading to resistance. Methods: Cell growth, proliferation, cell cycle phases, and cell cycle regulating proteins were compared in temsirolimus resistant (res) and sensitive (parental—par) RT112 and UMUC3 bladder cancer cells. To evaluate invasive behavior, adhesion to vascular endothelium or to immobilized extracellular matrix proteins and chemotactic activity were examined. Integrin α and β subtypes were analyzed and blocking was done to evaluate physiologic integrin relevance. Results: Growth of RT112res could no longer be restrained by temsirolimus and was even enhanced in UMUC3res, accompanied by accumulation in the S- and G2/M-phase. Proteins of the cdk-cyclin and Akt-mTOR axis increased, whereas p19, p27, p53, and p73 decreased in resistant cells treated with low-dosed temsirolimus. Chemotactic activity of RT112res/UMUC3res was elevated following temsirolimus re-exposure, along with significant integrin α2, α3, and β1 alterations. Blocking revealed a functional switch of the integrins, driving the resistant cells from being adhesive to being highly motile. Conclusion: Temsirolimus resistance is associated with reactivation of bladder cancer growth and invasive behavior. The α2, α3, and β1 integrins could be attractive treatment targets to hinder temsirolimus resistance.

## 1. Introduction

Bladder cancer represents the second most prevalent genitourinary malignancy [1] and is the ninth most common malignancy worldwide [2]. Current treatments for non-muscle-invasive disease confer a 5 years cancer-specific survival rate of about 90%. However, muscle-invasive, recurrent, or metastatic bladder cancer is associated with a poor prognosis [3,4,5].

First-line treatment of metastatic disease is commonly based on a cisplatin-containing chemotherapy, with MVAC (methotrexate, vinblastine, doxorubicin, cisplatin) or GC (gemcitabine, cisplatin) [6]. Although these agents have improved patient outcome, median survival is still limited to 14.0 (GC) or 15.2 months (MVAC) with a 5 years overall survival rate of 13.0% and 15.3%, respectively [7]. Following relapse, the median survival is only 5 to 7 months [8].

During the last years, several immune checkpoint inhibitors have been approved for patients with urothelial carcinoma [9]. Clinical trials point to improved outcomes of patients subjected to these agents as a post-platinum treatment option. However, the overall response rate has remained disappointingly low [9], leaving a great number of patients without profit from chemo- and/or immunotherapy [10].

Since over 40% of bladder cancers exhibit constitutive activation of the phosphatidylinositol 3-kinase/protein kinase B/mechanistic target of rapamycin (PI3K/AKT/mTOR) pathway [10,11], suppressing activation is a treatment option [12]. mTOR pathway activation has been shown to be closely involved in bladder cancer tumorigenesis and to be a predictor of disease progression and poor cancer specific survival [3,13,14,15]. Yuge et al. have provided evidence that activation of the PI3K/Akt/mTOR pathway in bladder cancer may be responsible for acquired chemoresistance towards cisplatin [16]. A subset of bladder cancer patients, refractory to first line platinum-based chemotherapy, has been reported to benefit from the mTOR-inhibitor, temsirolimus [17]. In unselected patients, the mTOR-inhibitor, everolimus, has demonstrated clinical activity as a first-line monotherapy in advanced biliary tract cancer [18]. Both temsirolimus and everolimus have already been approved for first- or later-line use in the treatment of patients with advanced renal cell carcinoma.

Nevertheless, although mTOR-inhibition has been shown to be useful in treating bladder cancer, the benefit is not as strong as expected. Presumably, chronic blockade of the mTOR pathway leads to undesired feedback loops, limiting the efficacy of mTOR-inhibitors [19]. The mode of action responsible for the drug non-responsiveness has not been evaluated in detail. This study was designed to investigate behavior and molecular alterations of bladder cancer cells driven to temsirolimus resistance.

## 2. Results

### 2.1. Temsirolimus Resistance Alters Cell Growth, Proliferation, and Cell Cycling

**Figure 1:**
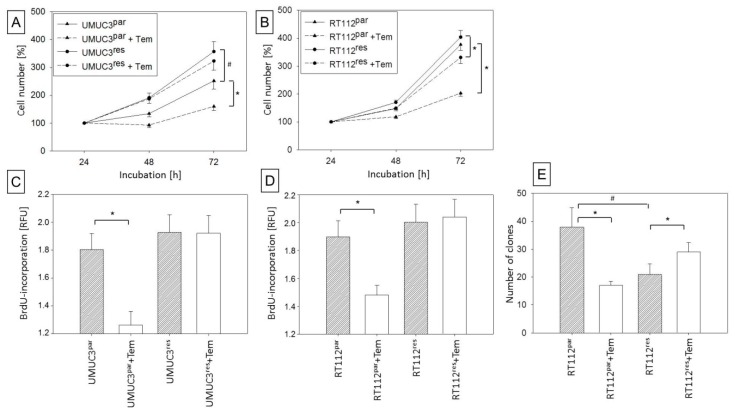
Growth of parental and temsirolimus-resistant bladder cancer cells. (**A**) UMUC-3 and (**B**) RT112 tumor cells exposed to 10 nmol/mL temsirolimus for 24 (100%), 48, and 72 h. Proliferation of parental and temsirolimus-resistant (**C**) UMUC3 and (**D**) RT112 exposed to temsirolimus [10 nmol/mL] during 48 h BrdU assay. (**E**) Clonal growth of RT112^par^ and RT112^res^ after 10 days incubation with temsirolimus [10 nmol/mL]. RFU = Relative Fluorescence Units. Bars indicate standard deviation (SD). * indicates significant difference to corresponding control, #indicates significant difference to parental control cells, *p* ≤ 0.05. *n* = 5.

Cell growth of RT112res, evaluated by the MTT cell growth assay, was similar to that of RT112par, while the cell number of UMUC3res even exceeded that of UMUC3par. Treatment of the UMUC3res cells with 10 nmol/mL temsirolimus revealed no significant growth impairment (Figure 1A), and a minor growth inhibition of RT112res (Figure 1B) occurred, compared to respective controls.

Since cell growth does not allow conclusions about the proliferative activity of the tumor cells, BrdU incorporation into cellular DNA during cell proliferation was also evaluated. Accordingly, proliferation of UMUC3par and RT112par was significantly diminished after exposure to temsirolimus, whereas UMUC3res and RT112res proliferation was not affected by temsirolimus, each compared to untreated controls (Figure 1C,D). A clone formation assay was performed to evaluate tumor cell propagation. Clonal growth of RT112par was significantly reduced, while clonal growth of RT112res was significantly elevated following temsirolimus application (Figure 1E). UMUC3 did not form clones and was therefore, not evaluated.

Apoptotic or necrotic events were not detected after temsirolimus treatment, indicating that reduced cell growth and proliferation were not caused by apoptosis or necrosis. Based on the drug sensitive UMUC3 cells, 1.88 ± 1.02% (control) versus 2.13 ± 1.78% (temsirolimus treatment) underwent early apoptosis, and 4.04 ± 3.72% (control) versus 3.28 ± 3.27% (temsirolimus treatment) were in late apoptosis. Early apoptosis of UMUC3res was 4.23 ± 3.84% (without temsirolimus re-treatment) versus 3.59 ± 2.88% (with temsirolimus re-treatment), and the percentage of UMUC3res in late apoptosis was 6.44 ± 3.88% (without temsirolimus re-treatment) versus 4.49 ± 2.41% (with temsirolimus re-treatment). Similar data were obtained for RT112 cells.

**Figure 2:**
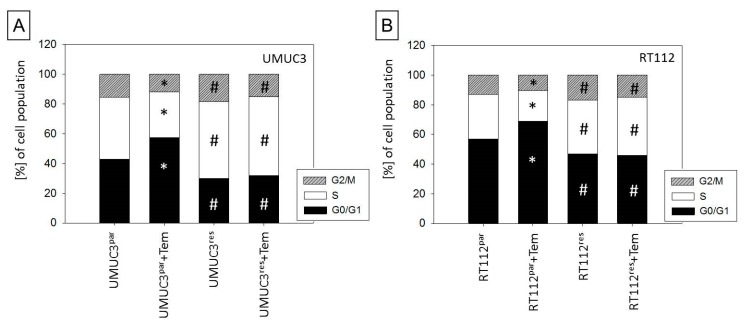
Cell cycle distribution following temsirolimus [10 nmol/mL] exposure. Percentage of parental and resistant (**A**) UMUC3 and (**B**) RT112 in G0/1, S, and G2/M phase is indicated. Controls remained untreated. One representative of three separate experiments is shown. * indicates significant difference to the controls. # indicates significant difference between res and par controls.

Since cell growth and proliferation is closely associated with cell cycle progression, the cell cycle phases of the treated tumor cells (versus controls) were subsequently analyzed. Cell cycle analysis demonstrated more resistant UMUC3 and RT112 cells to be in the G2/M- and S-phases, compared to respective parental cultures. The G0/G1-phase in parental UMUC3 and RT112 cells was up-regulated when treated with low-dosed temsirolimus, whereas treatment of both UMUC3res and RT112res with low-dosed temsirolimus provoked no response (Figure 2A,B).

Morphological differences between resistant and sensitive tumor cells were not observed.

### 2.2. Temsirolimus Resistance is Associated with Alterations of Cell Cycle Protein Expression

**Figure 3:**
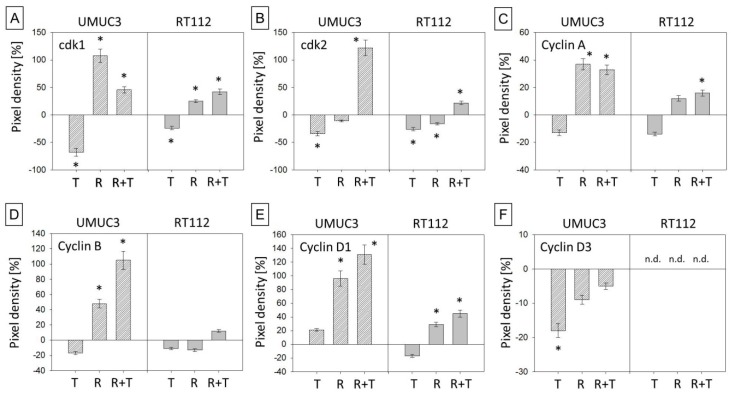

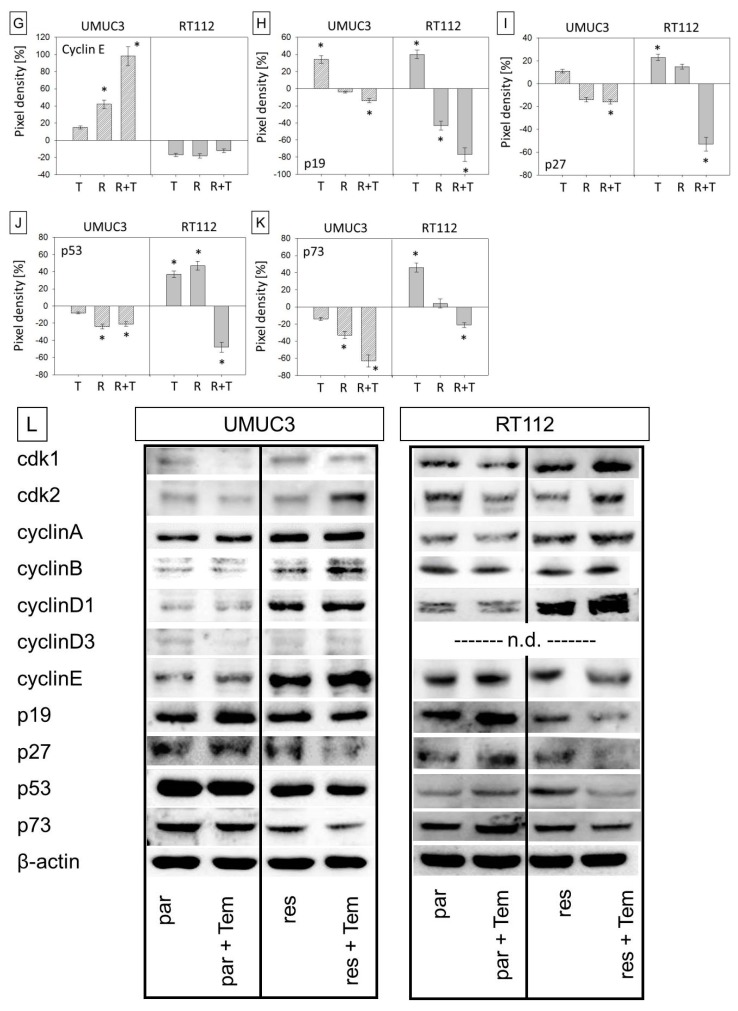
Protein expression profile of cell cycle regulating proteins. (**A**–**K**) Pixel density analysis of the protein expression in parental and temsirolimus-resistant UMUC-3 and RT112 cells after 72 h exposure to temsirolimus [10 nmol/mL]. All values are given in percentage difference to the parental control (set to 0). T = parental cells + temsirolimus, R = resistant cells, R + T = resistant cells + temsirolimus. Bars indicate standard deviation (SD). * indicates significant difference to parental control (set to 0), *p* ≤ 0.05. *n* = 4. n.d. = not detectable. β-actin served as internal control. (**L**) Original western blots: One representative of four separate experiments is shown. Each protein analysis was accompanied by a β-actin loading control. One representative internal control is shown here.

Since cell cycling is controlled by specific cell cycle regulating proteins, particularly cyclins, cylin-dependent kinases (cdk) and tumor suppressors of the p-family were analyzed. Cdk1 and 2 were reduced by temsirolimus in the parental but enhanced in the resistant tumor cells (Figure 3A,B and L). The cyclin members A, B, D1 and E were not modified by temsirolimus in parental cells but were enhanced in UMUC3res and RT112res (with a few exceptions, Figure 3C–E,G and L). In contrast, cyclin D3 was suppressed by temsirolimus in UMUC3par but not in UMUC3res (Figure 3F,L). Cyclin D3 was not detectable in RT112 cells. The regulatory elements p19 (Figure 3H,L; UMUC3 and RT112), p27, p53, and p73 (Figure 3I–L; RT112) increased in the parental cells, but were lost in UMUC3res and RT112res when treated with temsirolimus. 

### 2.3. Temsirolimus Modifies Akt-mTOR-Signalling

**Figure 4:**
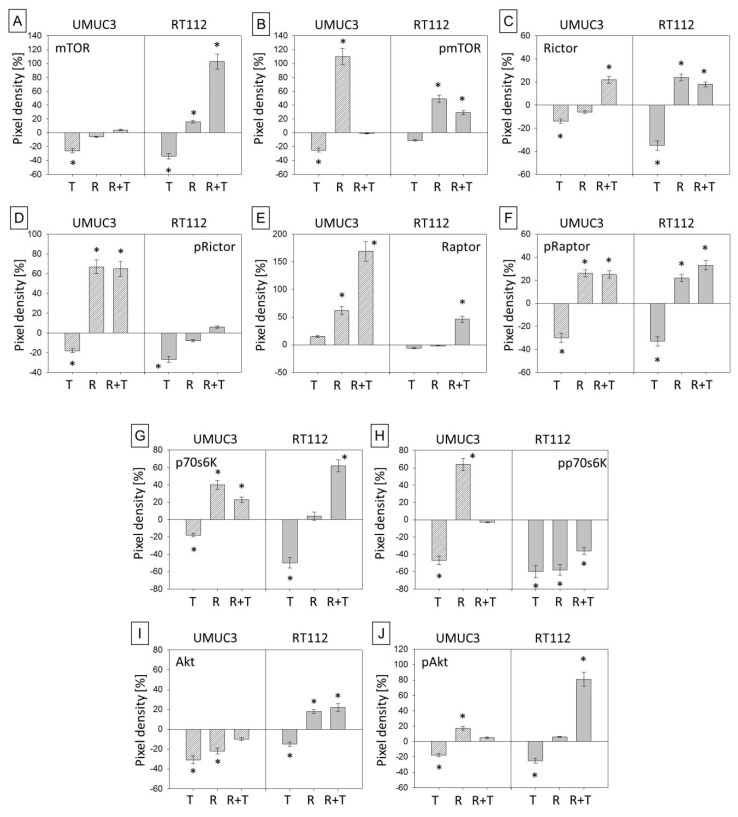

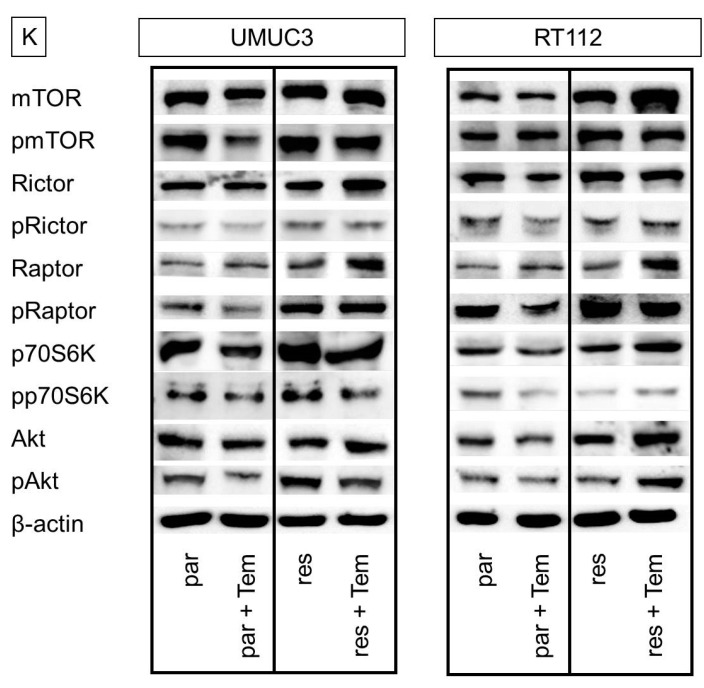
Protein expression profile of targeted and signalling proteins. (**A**–**J**) Pixel density analysis of the protein expression in parental and temsirolimus-resistant UMUC-3 and RT112 cells after 3 days exposure to temsirolimus [10 nmol/mL]. All values are given in percentage difference to the parental control (set to 0). T = parental cells + temsirolimus, R = resistant cells, R + T = resistant cells + temsirolimus. Bars indicate standard deviation (SD). * indicates significant difference to parental control (set to 0), *p* ≤ 0.05. *n* = 4. β-actin served as internal control. (**K**) Original western blots: One representative of four separate experiments is shown. Each protein analysis was accompanied by a β-actin loading control. One representative internal control is shown here.

Temsirolimus acts on the Akt-mTOR-signaling pathway. Therefore, modulation of the respective proteins was compared in resistant and sensitive tumor cells. UMUC3par and RT112par generally responded to temsirolimus in that mTOR (Figure 4A,K) and pmTOR (Figure 4B,K) and the sub-complexes Rictor (Figure 4C,K; along with pRictor, Figure 4D,K) and pRaptor (Figure 4E,K; along with pRaptor, Figure 4F,K) were down-regulated, whereas up-regulation was seen in the resistant cells re-exposed to temsirolimus. Both total and activated Akt (Figure 4I–K) and p70s6k (Figure 4G,H,K) were diminished in UMUC3par and RT112par cells by temsirolimus. In resistant cells, temsirolimus induced increased p70s6k expression in UMUC3res and RT112res (Figure 4G,K). Activated p70s6k was only moderately suppressed in RT112res, compared to RT112par (Figure 4H,K), and Akt and pAkt significantly increased in RT112res cells (Figure 4I–K).

### 2.4. Adhesion and Migration Properties of Resistant Versus Parental Cells

**Figure 5:**
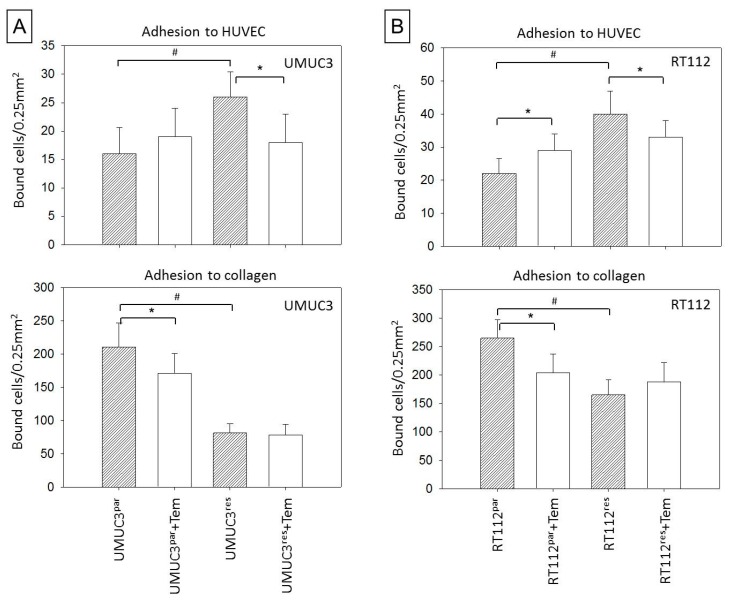
Adhesion of parental and temsirolimus-resistant (**A**) UMUC-3 and (**B**) RT112 to endothelium HUVEC (upper) and collagen (lower). Tumor cells were treated with 10 nmol/mL temsirolimus for 72 h. Controls remained untreated. Mean number of adherent tumor cells from five fields after 2 h (HUVEC) or 1/2 h (collagen) incubation. Bars indicate standard deviation (SD), * indicates significant difference to corresponding control, # indicates significant difference to parental control, *p* ≤ 0.05. *n* = 5.

Tumor progression is not only promoted by increased proliferation but also by increased spreading activity. Spreading requires the interaction of circulating tumor cells with the vessel endothelium and the underlying extracellular matrix (predominantly composed of collagen). Therefore, the influence of temsirolimus on tumor cell adhesion to endothelial cells (HUVEC) or an immobilized collagen matrix was explored to evaluate motile behavior (chemotaxis assay). Adhesion data were not uniform. Significantly more UMUC3res and RT112res attached to HUVEC, but significantly less UMUC3res and RT112res were bound to collagen when compared to the parental controls (Figure 5A,B). Following temsirolimus treatment, the attachment rate of RT112par to HUVEC increased, whereas UMUC3res and RT112res started to detach from the endothelial monolayer.

**Figure 6:**
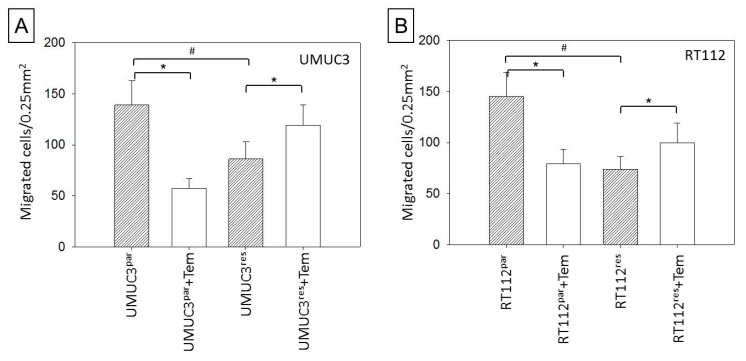
Effect of temsirolimus on chemotaxis in parental and temsirolimus-resistant (**A**) UMUC-3 and (**B**) RT112. Bladder cancer cells treated with 10 nmol/mL temsirolimus for 72 h. Chemotaxis through the membrane after 20 h was evaluated. Controls remained untreated. Bars indicate standard deviation (SD), * indicates significant difference to corresponding control, # indicates significant difference to parental control, *p* ≤ 0.05. *n* = 5.

Migration properties differed between parental and resistant tumor cell populations as well. The number of migrated parental cells exceeded that of resistant counterparts. However, exposure to temsirolimus caused significant suppression of UMUC3par or RT112par chemotaxis but a considerable increase of migrated UMUC3res and RT112res cells (Figure 6A,B).

### 2.5. Temsirolimus Resistance is Accompanied by an Altered Integrin Expression Profile

**Figure 7:**
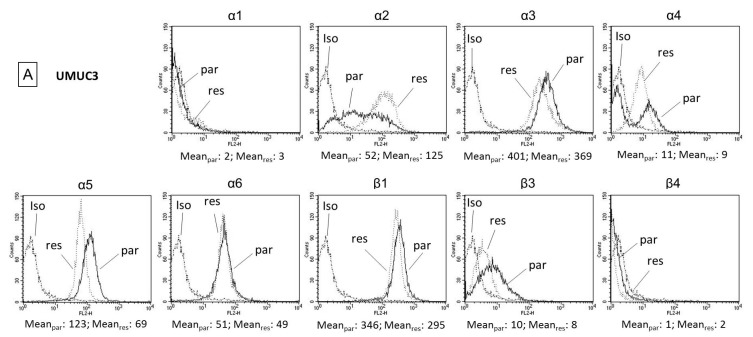

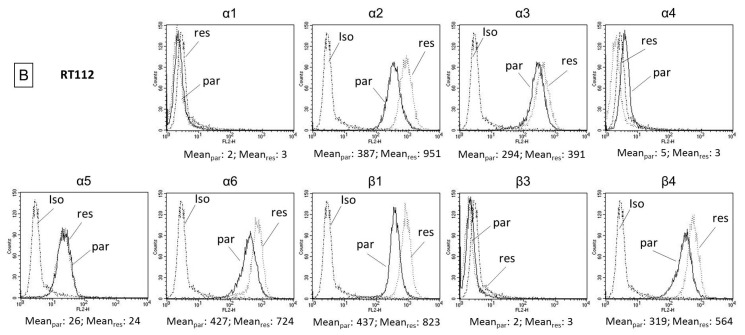
Surface expression of integrin subtypes and isotype control of (**A**) UMUC3par, UMUC3res and (**B**) RT112par, RT112res. Counts indicate MFI (mean fluorescence intensity). One representative of three separate experiments is shown.

Adhesion receptors of the integrin α- and β-family are involved in triggering tumor cell adhesion and migration. Consequently, integrin expression levels were analyzed by western immunoblot. UMUCpar strongly expressed the integrin members α3, α5, α6, and β1. The integrins α2, α4, and β3 were moderately expressed, and α1 and β4 were not detectable (Figure 7A). RT112par cells were characterized by a high α2, α3, α6, β1, and β4, and a moderate α5 expression level. The integrin subtypes α1, α4, and β3 were not present on the cell surface (Figure 7B). Temsirolimus resistance in UMUC3 cells caused distinct changes in the integrin expression pattern. Most importantly, α2 increased, whereas integrin α3, α5, and β1 decreased. Similar to UMUC3res, temsirolimus resistance in RT112res was associated with up-regulation of integrin α2. In contrast, integrins α3, α6, β1, and β4 in RT112res also increased while integrin α5 was not influenced.

**Figure 8:**
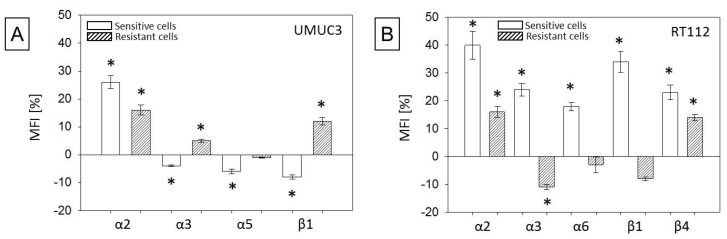
Surface integrin modification by temsirolimus. (**A**) UMUC3par and UMUC3res and (**B**) RT112par and RT112res. Corresponding controls were set to 0. Difference is presented as MFI [%]. Bars indicate standard deviation (SD), * indicates significant difference to corresponding control, *p* ≤ 0.05. *n* = 3.

Temsirolimus application to the parental and resistant cells caused elevation of α2 (UMUC3par > UMUC3res and RT112par > RT112res). An inverse behavior was seen with respect to integrin α3, α5, and β1 in UMUC3, in as much as α3, α5, and β1 were diminished in UMUC3par but α3 and β1 were enhanced in UMUC3res (Figure 8A). In RT112par, α3, α6, β1, and β4 increased in the resistant cells. In RT112res α3 was suppressed and β4 elevated (Figure 8B).

**Figure 9:**
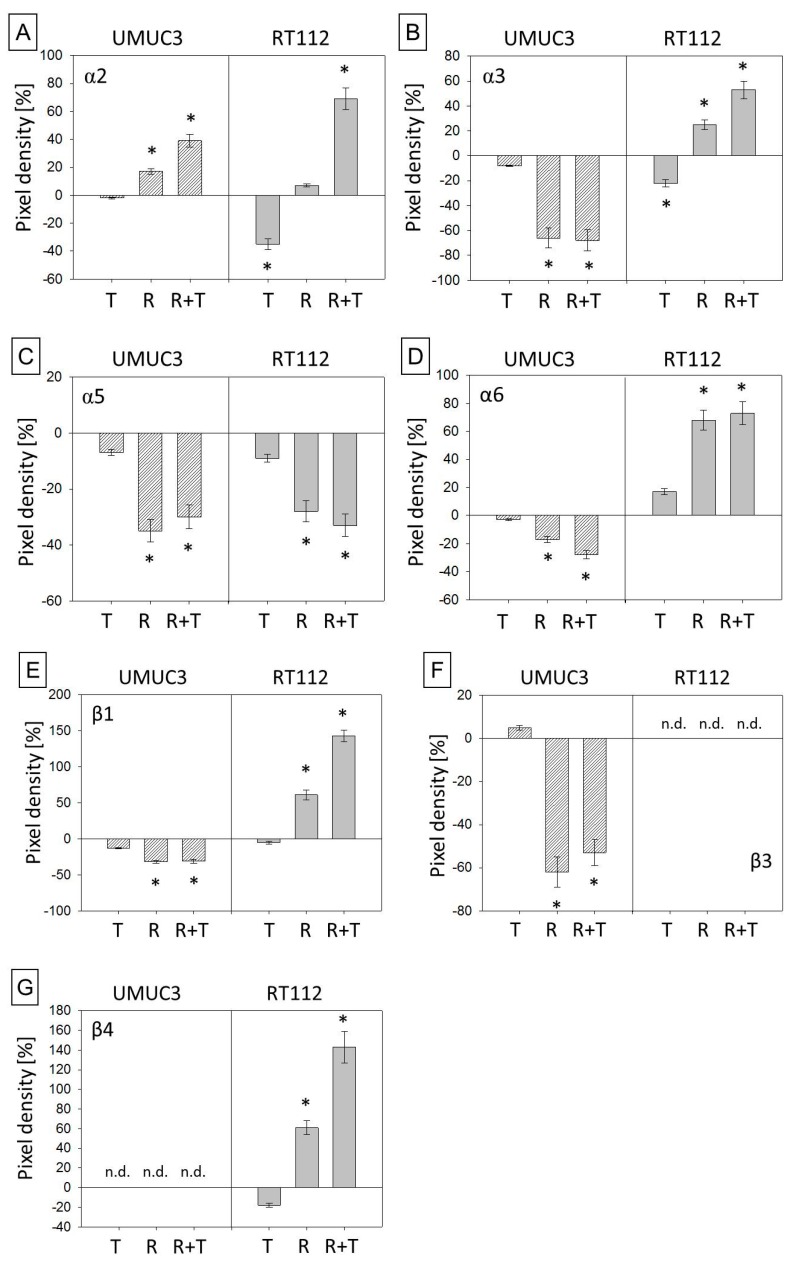

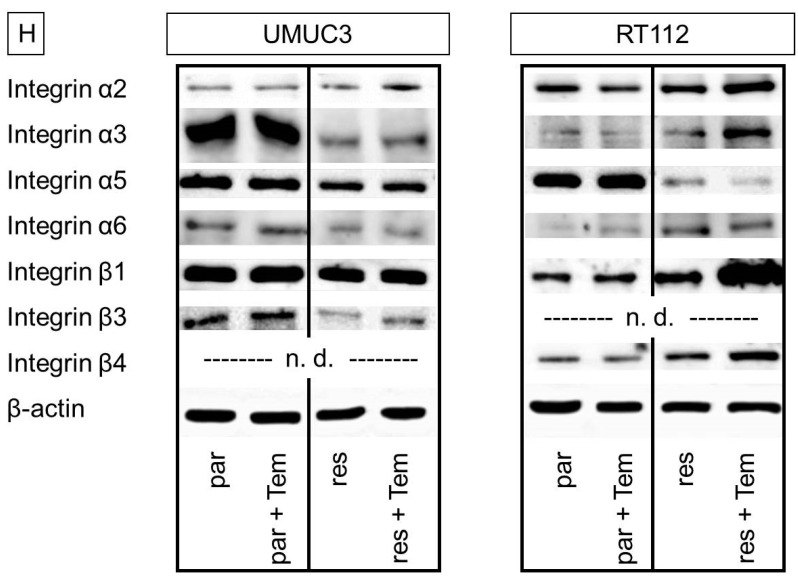
Total integrin content. (**A**–**G**) Pixel density analysis of the protein expression in parental and temsirolimus-resistant UMUC-3 and RT112 cells exposed to temsirolimus for 72 h. All values are given in percentage difference to the parental control (set to 0). T = parental cells + temsirolimus, R = resistant cells, R + T = resistant cells + temsirolimus. Differences to the parental control (set to 0) are shown. Bars indicate standard deviation (SD). * indicates significant difference to parental control, *p* ≤ 0.05. *n* = 4. β-actin served as internal control for western blotting. (**H**) Original western blots: One representative of four separate experiments is shown. Each protein analysis was accompanied by a β-actin loading control. One representative internal control is shown here.

Alterations in the intracellular integrin content are depicted in Figure 9. Analysis of UMUC3res documented enhanced α2 (Figure 9A,H) but lowered α3, α5, α6, β1, and β3 protein levels (Figure 9B–F,H) following temsirolimus exposure. Treatment of the parental cell line with temsirolimus did not cause significant integrin alterations compared to the untreated control. RT112res responded to temsirolimus such that α2, α3, α6, β1, and β4 increased (Figure 9A,B,D,E and G,H) and a5 decreased (Figure 9C,H).

### 2.6. Integrin Blocking Studies

**Figure 10:**
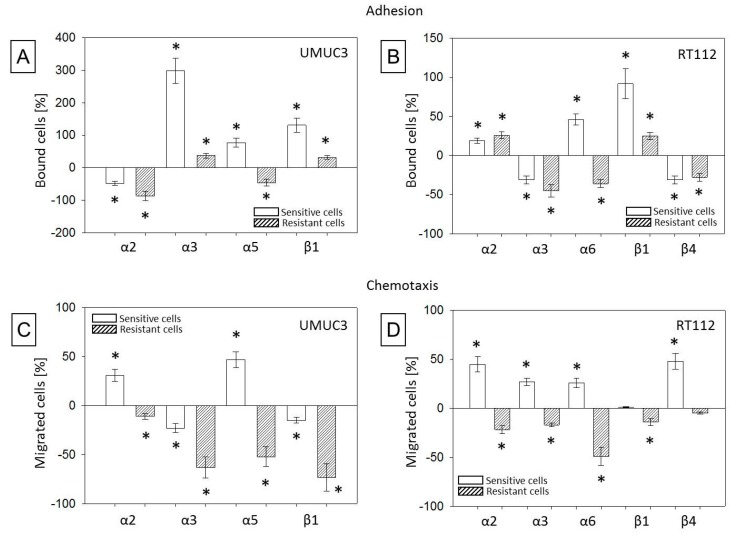
Influence of functional blocking of different integrin α and β subtypes on (**A**) UMUC3 and (**B**) RT112 cell adhesion to collagen as well as on (**C**) UMUC3 and (**D**) RT112 chemotaxis. Mean number of adherent or chemotactically active cells from five fields (0.25 mm^2^) were evaluated and percentage of blocked bladder cancer cells compared to the unblocked corresponding control cells (set to 0%,) was calculated. Bars indicate standard deviation (SD), * indicates significant difference to corresponding control, *p* ≤ 0.05. *n* = 5.

Blocking studies were carried out using function associated integrin antibodies directed towards α2, α3, α5, or β1 (UMUC3), or towards α2, α3, α6, β1, or β4 (RT112). The subtype members were chosen because their surface expression was distinctly modified by temsirolimus in the parental and resistant cell model. Integrin blockade differentially influenced the tumor cells’ adhesion behavior. Blocking α2 suppressed both UMUC3par and UMUC3res binding to immobilized collagen (Figure 10A). Blocking α3 or β1 led to a significant enhancement of tumor cell binding (UMUC3par > UMUC3res), and blocking α5 enhanced UMUC3par, but diminished UMUC3res adhesion.

Blocking β1 evoked similar effects on RT112 cells, however, an opposite reaction was seen when integrin α2 or α3 antibodies were applied, compared to UMUC3, evidenced by a decreased adhesion in the presence of the α2 but an increased attachment in the presence of the α3 antibody (Figure 10B). Blockade of the α6 receptor up-regulated RT112par adhesion but triggered loss of contacting RT112res, and β4 blockade induced reduced binding of both the parental and resistant cells.

Results of the chemotaxis study are notable inasmuch as an inverse behavior of RT112 cells was observed. Blocking α2, α3, α6, or β4 was associated with a significant activation of RT112par but a significant de-activation of RT112res motility (Figure 10C). The same effect was seen when UMUC3 were treated with the α2 or α5 antibody. On the other hand, blocking α3 or β1 universally diminished cell migration, whereby chemotaxis of UMUC3res was considerably more influenced than chemotaxis of UMUC3par (Figure 10D).

## 3. Discussion

Long-term application of temsirolimus to both RT112 and UMUC3 cells led to non-responsiveness, evidenced by growth and proliferation no longer being inhibited. Similarly, in kidney and prostate cancer cells, resistance towards the mTOR-inhibitor everolimus has been associated with increased mitotic activity [20,21]. Therefore, long-term suppression of mTOR could re-activate the mitotic machinery as evidenced by a profound increase in the S- and G2/M-phase in drug resistant bladder cancer cells.

Cell cycle progression is closely controlled by proteins of the cdk-cyclin family. In the present study, the cdk1-cyclin B- and the cdk2-cyclin A-complex were reduced in parental cells treated with temsirolimus, whereas a strong up-regulation was seen in drug resistant tumor cells, particularly when re-treated with temsirolimus. Li et al. recently reported that cell cycle progression of bladder cancer cells is driven by increased levels of cyclin B and cdk1 [22]. Enhanced activity of the cdk2-cyclin A axis is also closely associated with bladder cancer proliferation [23] with alterations of the cdk2 network as a key event during the process of resistance acquisition [24]. Therefore, up-regulation of cdk1/cyclin B and cdk2/cyclin A might be partly responsible for tumor progression after long-term temsirolimus exposure. Li et al. [22] and Tao et al. [25] have demonstrated that cdk1 depletion induces the expression of the tumor suppressors, p27 and p53. Loss of p27 and p53 was a typical feature of the drug resistant RT112 and UMUC3 cells, showing that elevating these proteins could counteract resistance associated tumor progression. Cyclin D1 functions as a key mitogen, with up to 20% of bladder cancer cases expressing an increase [26]. We also found a strong increase of cyclin D1 in temsirolimus resistant tumor cells accompanied by a considerable reduction of p73. In prostate cancer p73 has been shown to be a negative modulator of cyclin D1 [27]. Therefore, p73 down-regulation could account for the oncogenic activation of cyclin D1. Following temsirolimus application, cyclin D3 strongly decreased in parental but not in resistant UMUC3 cells while cyclin E was not reduced in parental tumor cells but was distinctly elevated in the resistant cells. The mTOR-inhibitor tacrolimus has been shown to induce a protective effect on bladder tumorigenesis by diminishing cyclin D3 and cyclin E [28]. Accordingly, cyclin D3 and E expression levels have been shown to closely correlate with bladder cancer recurrence and progression [29,30]. Based on the present investigation, we postulate that conversion from down- to up-regulation of cyclin D3 and E in the presence of temsirolimus might be a typical feature of resistance acquisition.

Following temsirolimus re-exposure, Akt phosphorylation increased in RT112res but not in UMUC3res. Wang et al. has proposed Akt activation occurring through a mechanism that depends on the rapamycin-sensitive mTOR complex 1 (mTORC1) but not on the rapamycin-insensitive mTORC2 [31]. This scenario might be true for RT112 cells since both Akt and Raptor (as a part of mTORC1), but not Rictor (as a part of mTORC2), increased under temsirolimus resistance. However, another mechanism should be considered for the resistant UMUC3 cells, where chronic temsirolimus application creates a feedback situation by inducing Raptor and Rictor activation as well. It is still difficult to explain why Akt was only slightly phosphorylated in the resistant cell subline and not activated at all when UMUC3res were treated with temsirolimus. The signaling mechanism of mTORC2 is distinct from that of mTORC1. mTORC1-Akt cross-communication is negatively associated, whereas activation of mTORC2 permanently elevates AKT phosphorylation by forming a positive feedback loop [32]. Presumably, activation of Raptor triggering Akt decrease and activation of Rictor triggering Akt increase may compensate each other in such a way that Akt remains unaltered. This issue requires further examination.

Beside tumor growth and proliferation, advanced cancer is characterized by distal metastases. More RT112res and UMUC3res attached to HUVEC in the present investigation, compared to parental cells, but lost endothelial contact following temsirolimus treatment. Diminished collagen binding of resistant cells was observed. In a recent evaluation of prostate cancer cells, loss of collagen-tumor cell binding was accompanied by increased laminin interaction as a prerequisite for metastatic progression [33]. We did not evaluate RT112 and UMUC3 binding to laminin. However, exposure of the drug resistant cell lines to temsirolimus resulted in a significant increase of chemotactic movement, whereas chemotaxis of the parental cell lines was suppressed. Akt/mTOR-signaling has been associated with migration and invasion of bladder cancer cells [34], whereby Rictor seems particularly involved in cytoskeleton organization and cell polarity [35,36]. Therefore, chronic use of temsirolimus may reactivate mTOR and not only accelerate bladder cancer cell proliferation but also force the tumor cell invasion cascade. In good corroboration, whole-exome and targeted sequencing in patients with aggressive bladder cancer has identified mTOR as an important target in treating patients with metastases [37].

Integrins and their associated signaling pathways have been shown to be closely involved in the metastatic progression of bladder cancer [38]. The data presented here demonstrate significant differences in the integrin expression pattern of parental cells and their resistant counterparts. Based on the UMUC3-model, the integrin members α2, α3, α5, and β1 were identified as relevant parameters. α5 surface expression was down-regulated by temsirolimus in the parental but not in the resistant cells, and α3 and β1 were suppressed in UMUCpar but elevated by temsirolimus in UMUCres. Simultaneously, the cytoplasmic protein level of α3 and β1 was reduced in UMUCres following temsirolimus re-exposure. At least these two receptor proteins seem to be translocated from the intracellular space to the outer cell surface. This phenomenon is not uncommon. The integrins α7 and β3 in renal cell carcinoma, and α5 and β4 in prostate cancer cells have been shown to be translocated when mTOR has been blocked [39,40,41]. Besides integrin redistribution, subtype function differed between parental and resistant tumor cells. Blockade of α5 led to up-regulation of UMUCpar adhesion and chemotaxis but to down-regulation of UMUCres adhesion and chemotaxis. The same was true with respect to α2 driven control of UMUC3 movement. The functional switch of particular integrin-subtypes seems to be a typical feature in the course of resistance acquisition and has been considered crucial to accelerating metastatic invasion [40,42]. Since α2, α3, and β1 expression negatively correlated with UMUC3res chemotaxis, we conclude that elevation of these integrins of the tumor cell surface, after adding temsirolimus in a clinically relevant dosage, serves as an important stimulus to form secondary tumors. Increased integrin α2 has been documented in metastatic lymph nodes and distant metastases in chemoresistant gastric cancer [43]. Analysis in TCGA datasets demonstrates that high levels of integrin α3 are closely associated with a poor prognosis for patients with metastasized colorectal cancer [44]. Finally, integrin β1 has been shown to confer cisplatin bevacizumab resistance and to be associated with metastatic formation [45,46]. The role of integrin α5 is not clear. It was not altered on the UMUC3res cell surface but was diminished inside the cell under temsirolimus re-exposure. Analysis of invasive urothelial bladder cancer samples demonstrated an enhanced α5 level, compared to normal urothelium [47]. In contrast to these findings, low α5 expression has been associated with high invasiveness of breast cancer cells. Furthermore, loss of this integrin resulted in growth activation of this tumor entity [48]. Whether reduced integrin α5 protein expression may activate bladder cancer growth as well remains uncertain. However, cross-communication between Rictor and α5 has been described [49], making a scenario likely where phosphorylation of Rictor in UMUC3res is coupled with the suppression of integrin α5. Still, this is speculative and requires further investigation.

Beside integrins α2, α3, and β1, the family members α6 (but not α5) and β4 were enhanced on the surface membrane of RT112res cells, compared to RT112par. Another resistance constellation, compared to that of UMUC3, must therefore be assumed for this cell line. Functional blocking of β4 reduced RT112res adhesion but did not influence chemotaxis. Blocking α2, α3, α6, or β1 all diminished chemotaxis but either enhanced adhesion (α2, β1) or lowered it (α3, α6). Resistance caused elevation of α2, and β1 forces RT112 adhesion and more cells are allowed to migrate. Elevation of α3 and α6 is interpreted such that the tumor cells are loosening a firm contact with the extracellular matrix to actively start invasion. The β4 integrin obviously exerts a predominant role in regulating tumor cell adhesion. In fact, over-expression of integrin β4 has been positively associated with Akt-mTOR signaling and lung metastases of hepatocellular carcinoma [50]. Since both Akt and β4 increased in RT112res, Akt driven elevation of β4 should be considered in this cell line.

## 4. Materials and Methods

### 4.1. Cell Cultures and Temsirolimus Treatment

RT112 and UMUC3 (ATCC/LGC Promochem GmbH, Wesel, Germany) bladder carcinoma cells were grown and subcultured in RPMI 1640 supplemented with 10% fetal calf serum (FCS), 20 mM HEPES-buffer, 1% glutamax and 1% penicillin/streptomycin (all: Gibco/Invitrogen; Karlsruhe, Germany) in a humidified, 5% CO_2_ incubator. RT-112 is an invasive (pathological stage T2) moderately differentiated (grade 2/3) model of human bladder cancer, UMUC-3 a high grade 3, invasive bladder cancer. The temsirolimus resistant sublines were established by exposing parental cells over 12 months to temsirolimus (Torisel^®^, LC Laboratories, Woburn, MA, USA), starting at 1 nmol/mL and increasing stepwise to 1 µmol/mL. The resistant sublines were termed UMUC3res and RT112res. The parental control cells, treated with culture medium alone, were designated UMUC3par and RT112^par^. Cell viability was determined by trypan blue (Gibco/Invitrogen).

To compare the influence of temsirolimus on the behavior of resistant and parental tumor cells, cell culture medium of UMUC3res or RT112res containing 1 μmol/mL temsirolimus was replaced by temsirolimus-free medium to avoid unspecific effects. A medium change was also carried out in the drug-sensitive cell culture system. After 72 h, a clinically relevant dosage of 10 nmol/mL temsirolimus was added to both resistant and parental cells (controls received fresh medium without temsirolimus). Cell cultures were then subjected to the experiments described below.

Endothelial cells were isolated from human umbilical veins (HUVEC) and harvested by enzymatic treatment with dispase (Gibco/Invitrogen). They were grown in Medium 199 (M199; Biozol, Munich, Germany), supplemented with 10% FCS, 10% pooled human serum, 20 mg/mL endothelial cell growth factor (Boehringer, Mannheim, Germany), 0.1% heparin, 100 ng/mL gentamycin, and 20 mM HEPES buffer (pH 7.4). Subcultures from passages 2–5 were employed.

### 4.2. Measurement of Tumor Cell Growth, Proliferation, and Apoptosis

Cell growth was assessed using the 3-(4,5-dimethylthiazol-2-yl)-2,5-diphenyltetrazolium bromide (MTT) dye reduction assay (Roche Diagnostics, Penzberg, Germany). Bladder cancer cells (50 µL, 1 × 10^5^ cells/mL) were seeded onto 96-well plates. After 24, 48, and 72 h, 10 µL MTT (0.5 mg/mL) were added for an additional 4h. Cells were then lysed in a buffer containing 10% SDS in 0.01 M HCl. The plates were incubated overnight at 37 °C, 5% CO_2_. Absorbance at 550 nm was determined for each well using a microplate enzyme-linked immunosorbent assay (ELISA) reader. After subtracting background absorbance, results were expressed as mean cell number.

Cell proliferation was measured using a BrdU cell proliferation enzyme-linked immunosorbent assay (ELISA) kit (Calbiochem/Merck Biosciences, Darmstadt, Germany). Tumor cells, seeded onto 96-well plates, were incubated with 20 µL BrdU-labeling solution per well for 8 h, fixed and detected using anti-BrdU mAb, according to the manufacturer’s instructions. Absorbance was measured at 450 nm using a microplate ELISA reader.

Clonal growth was evaluated through a clonogenic assay. 300 parental or temsirolimus resistant tumor cells were transferred into each well of 6-well plates. Both parental and resistant cells were exposed to temsirolimus [10 nmol/mL]. Untreated parental and resistant cells served as controls. After 10 days incubation colonies were fixed and counted. A colony consisted of at least 50 cells.

To evaluate apoptosis, the expression of Annexin V/propidium iodide (PI) was evaluated using the Annexin V-FITC Apoptosis Detection kit (BD Pharmingen, Heidelberg, Germany). Tumor cells were washed twice with PBS, and then incubated with 5 µL of Annexin V-FITC and 5 µL of PI in the dark for 15 min at RT. Cells were analyzed by flow cytometry using FACScalibur (BD Biosciences, Heidelberg, Germany). The percentage of apoptotic cells (early and late) in each quadrant was calculated using CellQuest software (BD Biosciences).

### 4.3. Analysis of Cell Cycling

Cell cycle analysis was carried out on cell cultures grown to subconfluency. Tumor cell populations were stained with PI, using a Cycle TEST PLUS DNA Reagent Kit (Becton Dickinson, city, state if USA, country) and then subjected to flow cytometry using FACScan (Becton Dickinson). A total of 10,000 events were collected from each sample. Data acquisition was carried out using CellQuest software and cell cycle distribution was calculated using the ModFit software (Becton Dickinson). The number of gated cells in G1-, G2/M- or S-phase was expressed as %.

### 4.4. Tumor Cell Adhesion to Vascular Endothelial Cells

To analyze tumor cell adhesion, HUVEC were transferred to 6-well plates in complete HUVEC medium. When confluent, UMUC3 and RT112 cells were detached from the culture flasks by accutase treatment (PAA Laboratories, Cölbe, Germany) and 0.5 × 10^6^ cells were then added to and left on the HUVEC monolayer for 2 h. Subsequently, non-adherent tumor cells were washed off using warmed (37 °C) PBS (Ca^2+^, Mg^2+^). The remaining cells were fixed with 2% glutaraldehyde. Adherent tumor cells were counted in five different fields of a defined size (5 × 0.25 mm^2^) using a phase contrast microscope and the mean cellular adhesion rate was calculated.

### 4.5. Attachment to Immobilized Extracellular Matrix Proteins

A total of 24-well plates were coated with collagen G (extracted from calfskin, consisting of 90% collagen type I and 10% collagen type III; Biochrom, Berlin, Germany; diluted to 400 μg/mL in PBS) overnight. Plastic dishes served as the background control. Plates were washed with 1% BSA (bovine serum albumin) in PBS to block nonspecific cell adhesion. 0.1 × 10^6^ tumor cells were then added to each well and left for 30 min incubation. Subsequently, non-adherent tumor cells were washed off, the remaining adherent cells were fixed with 2% glutaraldehyde and counted microscopically. The mean cellular adhesion rate, defined by adherent cells/coated well—adherent cells background, was calculated from five different observation fields (5 × 0.25 mm^2^).

### 4.6. Chemotactic Activity

Serum induced chemotactic movement was examined using 6-well Transwell chambers (Greiner, Frickenhausen, Germany) with 8-µm pores. 0.5 × 10^6^ UMUC3 or RT112 cells/mL were placed in the upper chamber in serum-free medium. The lower chamber contained 10% serum. After overnight incubation, the upper surface of the transwell membrane was gently wiped with a cotton swab to remove cells that had not migrated. Cells moving to the lower surface of the membrane were stained using hematoxylin and counted microscopically. The mean migration rate was calculated from five different observation fields (5 × 0.25 mm^2^).

### 4.7. Expression of Cell Cycle Regulating and Signalling Proteins

To explore cell cycle regulating proteins, tumor cell lysates were applied to a 7–15% polyacrylamide gel (depending on protein size) and electrophoresed for 90 min at 100 V. The protein was then transferred to nitrocellulose membranes (1 h, 100 V). After blocking with non-fat dry milk for 1h, the membranes were incubated overnight with monoclonal antibodies directed against the following cell cycle proteins, all from BD Biosciences, Heidelberg, Germany: Cdk1 (IgG1, clone 1, dilution 1:2500, 34 kDa), cdk2 (IgG2a, clone 55, dilution 1:2500, 33 kDa), cyclin A (IgG1, clone 25, dilution 1:250, 60 kDa), cyclin B (IgG1, clone 18, dilution 1:1000, 62 kDa), cyclin D1 (IgG1, clone G124-326, dilution 1:250, 36 kDa), cyclin D3 (IgG2b, clone 1, dilution 1:1000, 33 kDa), cyclin E (IgG1, clone HE12, dilution: 1:1000, 50 kDa), Skp1 p19 (IgG1, clone 52/p19, dilution 1:5000, 19 kDa), p27 (IgG1, clone 57, dilution 1:500, 27 kDa), p53 (IgG2b, clone Do-7, dilution: 1:1000, 53 kDa), p73 (IgG1, clone ER-15, dilution:1:1000, 73 kDa).

To evaluate mTOR, the following monoclonal antibodies were employed: Anti-mTOR (IgG, clone 7C10, 289 kDa), anti-phospho mTOR (pmTOR; IgG, Ser2448, clone D9C2, 289 kDa), anti-Rictor (IgG, clone D16H9, 200 kDa), anti-phospho Rictor (IgG, Thr1135, clone D30A3, 200 kDa), anti-Raptor (IgG, clone 24C12, 150 kDa) anti-phospho Raptor (IgG, Ser792, 150 kDa), anti-p70S6 Kinase (p70S6k; rabbit, IgG, clone 49D7, 70 kDa), anti-phospho p70S6k (pp70S6k, IgG, Thr389, clone 108D2, 70 kDa, all: New England Biolabs, Frankfurt, Germany, dilution 1:1000), Akt (clone 55, 60 kDa), and anti-phospho Akt (pAkt, pS472/pS473, clone 104A282, 60 kDa, both: IgG1, BD Biosciences, Heidelberg, Germany, dilution 1:500).

HRP-conjugated goat-anti-mouse IgG (Upstate Biotechnology, Lake Placid, NY, USA; dilution 1:5000) served as the secondary antibody. The membranes were briefly incubated with ECL detection reagent (ECLTM, Amersham/GE Healthcare, München, Germany) to visualize the proteins and then analyzed by Fusion FX7 system (Peqlab, Erlangen, Germany). β-actin (1:1000, 42 kDa; Sigma, Taufenkirchen, Germany) served as the internal control.

Gimp 2.8 software was used to perform pixel density analysis of the protein bands. The ratio of protein intensity/β-actin intensity was calculated, and expressed in percentage, related to controls set to 100%.

### 4.8. Integrin Surface and Protein Expression

Tumor cells were washed in blocking solution (PBS, 0.5% BSA) and then incubated for 60 min at 4 °C with phycoerythrin (PE)-conjugated monoclonal antibodies directed against the following integrin subtypes: Anti-α1 (mouse IgG1; clone SR84), anti-α2 (mouse IgG2a; clone 12F1-H6), anti-α3 (mouse IgG1; clone C3II.1), anti-α4 (mouse IgG1; clone 9F10), anti-α5 (mouse IgG1; clone IIA1), anti-α6 (mouse IgG2a; clone GoH3), anti-β1 (mouse IgG1; clone MAR4), anti-β3 (mouse IgG1; clone VI-PL2), or anti-β4 (rat IgG2b; clone 439–9B; all: BD Pharmingen, Heidelberg, Germany). Integrin expression of tumor cells was then measured using a FACscan (BD Biosciences, Heidelberg; FL-2H (log) channel histogram analysis; 1 × 10^4^ cells/scan) and expressed as mean RFI (relative fluorescence intensity). Mouse IgG1-PE (MOPC-21), IgG2a-PE (G155–178) and rat IgG2b-PE (R35-38; all: BD Biosciences) were used as isotype controls.

Integrin protein expression was evaluated by western blotting as described above, using the following antibodies: integrin α1 (rabbit, polyclonal, Chemicon/Millipore GmbH, Schwalbach, Germany, dilution 1:1000, 200 kDa), integrin α2 (mouse IgG1, clone 2; BD Biosciences, dilution 1:250, 150 kDa), integrin α3 (rabbit, polyclonal, Chemicon/Millipore, Schwalbach, Germany, dilution 1:1000, 150 kDa), integrin α4 (mouse, clone: C-20, Santa Cruz Biotechnology (USA), dilution 1:200, 150 kDa), integrin α5 (mouse IgG2a, clone 1; BD Biosciences, dilution 1:5000, 150 kDa), integrin α6 (rabbit, clone H-87, Santa Cruz Biotechnology (USA), dilution 1:200, 150 kDa), integrin β1 (mouse IgG1, clone 18; BD Biosciences, dilution 1:2500, 130 kDa), integrin β3 (mouse IgG1, clone 1; BD Biosciences, dilution 1:2500, 104 kDa), and integrin β4 (mouse IgG1, clone 7; BD Biosciences, dilution 1:250, 200 kDa).

### 4.9. Blocking Studies

UMUC3 and RT112 cells were incubated for 60 min with 10 μg/mL function-blocking anti–integrin α2 (clone P1E6), anti-integrin α3 (clone P1B5), anti–integrin α6 (clone NKI-GoH3), anti-integrin β1 (clone 6SG), or anti-integrin β4 (clone ASC-8; all: MerckMillipore). Controls were incubated with cell culture medium alone. Subsequently, tumor cell adhesion to immobilized collagen as well as chemotaxis was analyzed as described above.

### 4.10. Statistics

All experiments were performed 3–6 times. Statistical significance was determined by the Wilcoxon–Mann-Whitney-*U*-test. Differences were considered statistically significant at a *p* value < 0.05.

## 5. Conclusions

Chronic treatment of the bladder cancer cell lines RT112 and UMUC3 with the mTOR-inhibitor temsirolimus triggers resistance characterized by accelerated tumor growth and invasive behaviour. Temsirolimus reactivates the Akt/mTOR-pathway with down-regulation of the tumor suppressor proteins p27, p53, and p73, making elevation of these proteins a possible treatment target directed towards growth inhibition. Temsirolimus resistance with increased motility is associated with integrin translocation from the cytoplasm to the cell surface. The integrins α2, α3, and β1 were translocated to the surface of both the temsirolimus resistant cell lines UMUC3 and RT112. In resistant RT112 the α6 and β4 integrins were translocated as well. The lack of congeneric integrin response suggests that treatment targeting the integrins of different tumor types may also have to be different.
