# Expression of Concern: Juengel et al. Mechanisms Behind Temsirolimus Resistance Causing Reactivated Growth and Invasive Behavior of Bladder Cancer Cells In Vitro. *Cancers* 2019, *11*, 777

**DOI:** 10.3390/cancers18071077

**Published:** 2026-03-26

**Authors:** Cancers Editorial Office

**Affiliations:** MDPI, St. Alban-Anlage 66, 4052 Basel, Switzerland; cancers@mdpi.com

The Editorial Office of the *Cancers* journal would like to inform readers of concerns regarding apparent image duplication identified within Figure 4K of this article [1]. An assessment was conducted by the Editorial Board in consultation with the authors; however, as the original image data are no longer available for verification, the concerns could not be resolved. This notice is issued to ensure transparency and to maintain the integrity of the scholarly record. No further editorial action is planned at this time. The journal will reconsider this decision should additional information become available.
